# The Impact of Augmented Information on Visuo-Motor Adaptation in Younger and Older Adults

**DOI:** 10.1371/journal.pone.0012071

**Published:** 2010-08-09

**Authors:** Mathias Hegele, Herbert Heuer

**Affiliations:** IfADo - Leibniz Research Centre for Working Environment and Human Factors, Dortmund, Germany; Kyushu University, Japan

## Abstract

**Background:**

Adjustment to a visuo-motor rotation is known to be affected by ageing. According to previous studies, the age-related differences primarily pertain to the use of strategic corrections and the generation of explicit knowledge on which strategic corrections are based, whereas the acquisition of an (implicit) internal model of the novel visuo-motor transformation is unaffected. The present study aimed to assess the impact of augmented information on the age-related variation of visuo-motor adjustments.

**Methodology/Principal Findings:**

Participants performed aiming movements controlling a cursor on a computer screen. Visual feedback of direction of cursor motion was rotated 75° relative to the direction of hand motion. Participants had to adjust to this rotation in the presence and absence of an additional hand-movement target that explicitly depicted the input-output relations of the visuo-motor transformation. An extensive set of tests was employed in order to disentangle the contributions of different processes to visuo-motor adjustment. Results show that the augmented information failed to affect the age-related variations of explicit knowledge, adaptive shifts, and aftereffects in a substantial way, whereas it clearly affected initial direction errors during practice and proprioceptive realignment.

**Conclusions:**

Contrary to expectations, older participants apparently made no use of the augmented information, whereas younger participants used the additional movement target to reduce initial direction errors early during practice. However, after a first block of trials errors increased, indicating a neglect of the augmented information, and only slowly declined thereafter. A hypothetical dual-task account of these findings is discussed. The use of the augmented information also led to a selective impairment of proprioceptive realignment in the younger group. The mere finding of proprioceptive realignment in adaptation to a visuo-motor rotation in a computer-controlled setup is noteworthy since visual and proprioceptive information pertain to different objects.

## Introduction

A large body of research on visuo-motor plasticity deals with adaptation to visuo-motor rotations. Classical studies used wedge prisms to induce shifts in visual direction [1]–[2]. Typically, participants in those experiments initially point to the optically displaced target location. In the course of adaptation, pointing movements are gradually modified so that the target location is reached and the discrepancy between the optically displaced target and the optically displaced pointing hand is minimized. Thus there is an adaptive shift of the direction of pointing, which more or less compensates the optical displacement. For example, for a target, which is optically displaced to the right, the adaptive shift in pointing direction is to the left. When the displacing wedge prism is removed, there is typically an aftereffect, which is generally referred to as a negative aftereffect as it is in the direction opposite to the optical displacement. It is, however, in the same direction as the adaptive shift and can thus be conceived as a part of this shift, which persists even though it is no longer adaptive.

In studies that used a prismatically induced shift of visual direction, the moving limb itself is optically displaced, so that there is a discrepancy between its visual and proprioceptive localization. Recent studies of visuo-motor adaptation have often used a remotely controlled cursor in a computer setup to introduce changes in visuo-motor mapping. With respect to such extrinsic transformations, for which the input is given by the location of the hand or another body part and the output by the location of an object such as a cursor on a computer monitor, those studies demonstrated that humans are able to adapt both to novel visuo-motor gains, i.e. novel ratios of visually perceived distances and the associated amplitudes of body movements [3]–[5], and novel visuo-motor rotations, i.e. altered relations between the visually perceived direction of a target and the associated movement of an effector [5]–[10]. Moreover, adjustment to extrinsic visuo-motor transformations was shown to suffer in older adults above retirement age (mean age of 64 years and above [11]–[15]) and below retirement age (mean age of 56 years [16]–[18]) provided that the transformations are sufficiently complex, comprising, for instance, a nonlinear gain [17] or a direction-dependent gain [18] or a sufficiently large rotation [16].

The present study builds on previous work on age-related differences in adjusting to a single visuo-motor rotation [16] in order to explore the impact of additional visual cues on implicit and explicit knowledge contributing to visuo-motor adjustment. By implicit knowledge we refer to an internal model that approximates the respective transformation [19]–[20], automatically assigning movement parameters appropriate to reach the visual target. Implicit knowledge is indexed by aftereffects when participants are aware that a previously practiced visuo-motor transformation has been removed. By explicit knowledge we refer to conscious awareness of the characteristics of the transformations, which, in conjunction with contingent contextual cues, can serve as a basis for strategic corrections [21]. During practice of a novel visuo-motor transformation, participants can become aware of the nature of the transformation. They can subsequently use this knowledge to intentionally modify their movements in a feedforward fashion to compensate for the altered visuo-motor relationship.

A couple of empirical observations strongly suggest that age-related changes of adjustment to visuo-motor rotations primarily pertain to the generation of explicit knowledge along with the resultant strategic corrections, whereas implicit knowledge in terms of the development of an internal model is unaffected by age. Firstly, age-related differences are reported whenever the visuo-motor rotation was present and both, explicit and implicit knowledge, could contribute to performance. In contrast, no age-related differences are found in aftereffect measures, in which external cues indicate the absence of the transformation so that performance should not be affected by intentional strategic corrections [11]–[14], [16], [18]. Secondly, independently assessed explicit knowledge declines at older age [11], [16], [18], [21]. Thirdly, individual variations of explicit knowledge exhibit a systematic relation to individual variations of performance in the presence of the transformation, but not in its absence (aftereffects). When older and younger adults are matched by explicit knowledge, the age-related variation of visuo-motor adjustment disappears [16], [18], [21]. Finally, when the visuo-motor rotation is introduced incrementally in small steps outside of participants' awareness, age-related differences are absent even in the presence of the rotation [13]. Based on the findings described above, it seems reasonable to conclude that overall visuo-motor adjustment is stronger in younger than in older adults, supposedly due to age-related differences in the acquisition of explicit knowledge and/or application of deliberate strategic corrections based on such knowledge [16], [18], [21].

In the present study we made an attempt to boost the acquisition of explicit knowledge of the visuo-motor transformation, in particular in the older participants. A transformation is defined as a certain relation of an output signal to an input signal. Thus, awareness of both the output and the input signal should imply awareness of the relation, at least when the relation is as simple as a rotation. In the case of the visuo-motor rotation the output is the direction of cursor motion, which is consciously monitored by participants. The input is the direction of hand movement, which may be largely unnoticed, perhaps less so in younger than in older participants. Thus enhancing awareness of the hand movement appears as a feasible means to support the acquisition of explicit knowledge. A straightforward way to do so is to present the target for the hand movement (target input of the transformation) in addition to the target for the cursor motion (target output of the transformation). This is similar to the procedure of Mazzoni and Krakauer [22] who presented several targets with 45° separations simultaneously and instructed participants to move the hand to a target adjacent to the cursor target to compensate a visuo-motor rotation of 45°.

The augmented information about the visuo-motor transformation is expected not only to enhance explicit knowledge, perhaps more so in older than in younger participants, but also to enhance performance while it is present. However, it is not fully clear whether the induced intentional corrections might also affect implicit adjustments as they are reflected in the aftereffects. Even though implicit and explicit adjustments to visuo-motor transformations are functionally independent in principle [23], there can be interactions when the one kind of adjustment serves to change the informational basis for the other kind of adjustment. For example, when strategic corrections, based on explicit knowledge of the transformation, serve to reduce pointing errors and these errors contribute to implicit adjustments like the acquisition of an internal model of the transformation, aftereffects should be reduced as a consequence of strategic corrections. In fact, in prism-adaptation studies reduced aftereffects were observed in participants who had been aware of the visuo-motor transformation [24]–[25], whereas increased aftereffects were observed when participants were unaware of the transformation, e.g. due to an incremental introduction of the visual shift [26] or to damage to the parietal lobe in a condition called unilateral spatial neglect [27]. On the other hand, Mazzoni and Krakauer [22] observed an overcompensation of the visuo-motor rotation. They argued that implicit adjustments were superposed on the instructed intentional corrections. Therefore intentional corrections may not necessarily modify the informational basis for implicit adjustments.

In order to more thoroughly investigate the effects of augmented visual information on implicit visuo-motor adjustments, we also assessed visual shifts and proprioceptive shifts, which have been hypothesized to add up to the total aftereffect observed for pointing in studies of prism-adaptation [28]. Visual shifts are typically measured as changes of straight-ahead judgments of a visual stimulus after a period of prism adaptation, whereas proprioceptive shifts are measured as changes of straight-ahead pointing [29]. The relative size of these two kinds of adaptive changes depends on whether proprioceptive or visual information is more attended during adaptation, with the less attended modality being more affected [30]–[33].

In the present study we modified an experimental task with which we have shown age-related variations of visuo-motor adaptation that primarily pertained to explicit knowledge [16]. In order to compensate this effect of aging, we presented a hand target in addition to the cursor target during practice with a 75° rotation of visual feedback in the clockwise direction (CW). Accordingly, the hand target was rotated 75° in the counterclockwise direction (CCW) relative to the cursor target. The relation between these targets served to make the nature of the visuo-motor transformation quite obvious. On the second day there was a control condition in addition, in which there was no additional hand target during practice. The visuo-motor rotation was 75° CCW. This control only served to confirm our previous findings on adaptation to a 75° rotation [16]. It was intended for qualitative comparisons with the augmented information condition, but not for quantitative ones.

As in the previous study [16], we used a set of tests to assess different components of adaptation to the visuo-motor rotation. In particular these were visual open-loop tests in which the presence or the absence of the visuo-motor rotation was cued. They served to assess adaptive shifts and aftereffects, respectively. In addition we obtained explicit judgments of the directions of hand movements appropriate to reach different cursor targets in the presence of the rotation. To these tests we added visual-shift and proprioceptive-shift tests to assess the influence of the augmented information on two components that had been shown to contribute to aftereffects in prism-adaptation [34]–[35]. Visual-shift tests required the participants to match a line – more precisely: the endpoints of a line – to the horizontal or vertical. Proprioceptive-shift tests required the participants to move the hand repeatedly in the forward-backward or the left-right direction.

## Methods

### Participants

Two groups of participants were studied in this experiment. The younger participants, 9 men and 9 women, were 21 to 29 years old (mean: 24.1 years; SD: 2.4 years). The older participants, 10 men and 8 women, were 51 to 67 years old (mean: 59.2 years; SD: 4.3 years). The younger participants were students of Dortmund University, whereas most of the older participants responded to a newspaper ad. All participants were self-declared right-handers with normal color vision according to the Ishihara test [36]. The data of five additional participants were not included in the analyses. While two of them did not finish the experiment, data of three participants were excluded because for them there was at least one of the experimental conditions in which no regular trial was left after screening (see Data Analysis for a description of the screening procedure).

The study was conducted in accordance with the ethical standards laid down in the 1964 Declaration of Helsinki. All participants had given their informed consent in written form prior to the start of the experiment.

The older and younger participants were compared on a number of cognitive and sensorimotor tests prior to the experiment in order to establish that they were representative for their respective age groups in terms of typical age-related variations and invariances. The means and standard deviations are shown in Table 1. Conforming to typical findings [37], performance of the older participants was worse than that of the younger participants on the Digit Symbol Test of the German version of the Wechsler Adult Intelligence Scale [38], reflecting an age-related decline of fluid intelligence, but not on the Vocabulary Test, indicating age-related invariance of crystallized intelligence. In order to scan our participants for severe visuo-spatial and motor deficits that could influence the results on the adaptation task, we used a test of mental rotation ("Würfelaufgaben" of the IST, a German test of intelligence [39]), and a series of motor tests (subtests of the "Motorische Leistungsserie", all performed with the right hand; Schuhfried GmbH, Mödling, Austria; [40]). Among those tests, some significant age differences appeared. Older participants exhibited poorer performance in the mental rotation test and slower performance in the aiming test with aiming errors being of similar frequency across groups. For the pegboard test, the slowing only approached statistical significance. Furthermore, older adults produced more errors in the tracing test. Contrary to typical findings [41], however, the tracing test was performed faster by the older than by the younger group, a finding that we have observed repeatedly [42]. Finally, older and younger adults did not differ with respect to the number of taps generated during a fixed period of time, whereas the former produced more errors during a test of hand steadiness. However, this difference only apporached significance.

**Table 1:** Comparison of the younger and older adults on a set of control variables.

	young	old	Mann-Whitney-U
Digit Symbol	64.5 (13.2)	46. 1 (12.7)	U(18,16) = 54, p<.01
Vocabulary	23.8 (3.4)	21.7 (4.3)	U(18,18) = 122, p>.2
Mental Rotation	11.3 (3.5)	8.1 (2.8)	U(18,18) = 87.5, p<.05
Aiming: duration	7.9 (1.8)	10.1 (2.2)	U(11,16) = 35, p<.01
Aiming: errors	0.9 (2.2)	0.6 (1.3)	U(11,16) = 82, p>.7
Pegboard: duration	35.2 (3.2)	40.2 (6.5)	U(11,16) = 50, p<.1
Tracing: errors	17.7 (5.6)	23.9 (7.6)	U(11,16) = 45, p<.05
Tracing: duration	34.6 (7.8)	28.5 (6.1)	U(11,16) = 46, p<.05
Tapping: # of taps	202.5 (15.4)	198.4 (15.3)	U(11,16) = 67, p>.2

For each group the means and the standard deviations (in brackets) are given, and for each variable the result of a Mann-Whitney U-test (durations are in s). In the cognitive tasks, higher values indicate better performance.

### Apparatus

Participants sat on a height-adjustable chair and faced a 15-inch LCD monitor (EIZO FlexScan L365), which was placed in about 100 cm distance from their eyes on a table platform. Between the monitor and the participants a glass plate was placed on the table on which the movements were performed. The right index finger of the participants was strapped to a slide of 50 mm×30 mm (6 mm height), which ran on the glass plate with only little friction. Located directly above the fingernail, the slide carried a vertically oriented sensor of a miniBIRD system (miniBIRD 800, Ascension Technology Corporation). The position of the finger was recorded at 103.6 Hz (spatial resolution: 0.11 mm). An occluder 20 cm above the table platform prevented vision of the hand. The experiment was controlled via MATLAB and the Psychophysics Toolbox [43]–[44] on a Fujitsu Siemens workstation PC equipped with a Pentium 4 CPU running at 3 Ghz, 1 GB of RAM, and an ATI Radeon 9250 SE GPU with 128 MB memory.

### Task

Participants had to produce aimed movements to targets in different directions from a common start location centered on the screen. On the table surface the start was about 30–40 cm in front of the participant (18.5 cm from the edge of the table) and laterally displaced from the median plane by about 14 cm. The start location was the same in all trials, but the target location varied. The target amplitude was 90 mm, the target direction could be 0°, 45°, 90°, 135°, 180°, 225°, 270°, or 315° (0° is from the start location to the right). The start location was marked on the monitor by an outline circle of 9.6 mm diameter. A filled white circle of 5.6 mm diameter marked the respective target location in each trial. The current finger location was indicated on the monitor by the location of a cursor, a filled circle of 4.8 mm diameter. In closed-loop trials the cursor was visible during the movement, but not in open-loop trials. Participants were instructed to move swiftly and as accurately as possible.

In the present study the amplitude of the hand movement was identical to the amplitude of the cursor motion (visuo-motor gain of 1). However, the mapping of the direction of the hand movement on the direction of the cursor motion was varied. In the baseline condition the direction of the cursor motion was the same as the direction of the hand movement. In the rotation-on conditions, a visuo-motor rotation was in effect rotating the direction of the cursor motion 75° clockwise or counterclockwise relative to the direction of the hand movement.

Throughout the experiment, participants were instructed that there were trials with and without a novel visuo-motor rotation. Upon the start of the practice phase on the first day of the experiment, participants were informed about the presence of a transformation and told that they would see a second target. This second target was a yellow-colored hand icon. Participants were instructed that this icon represented the location to which they would have to move their hand in order to move the cursor to the respective cursor target. Absence or presence of the transformation was cued by the color of the start circle, red meaning *rotation on* and green *rotation off*, respectively.

### Design and procedure

The first day of the experiment consisted of four phases, baseline practice, pretests, practice with a visuo-motor rotation of 75° CW, and posttests. The experiment started with three blocks of baseline practice, each block consisting of 48 visual closed-loop trials with a target amplitude of 90 mm. In each block there were six random permutations of the 8 target directions without any target direction being repeated in successive trials. During all baseline practice trials, pretest trials, and associated maintenance trials the start circle was green to cue the absence of the visuo-motor rotation, and no additional hand target was presented.

Pretests were a visual open-loop test without transformation, an explicit test, a test of proprioceptive shift, and a test of visual shift in this order. The open-loop test consisted of three blocks of 8 trials each, with each target direction occurring once. Each test block was preceded by 8 maintenance trials that were identical to baseline practice trials.

The subsequent explicit test consisted of two blocks of 8 trials each, again each target direction occurring once per block and each block being preceded by maintenance trials. Each trial began with the presentation of the start circle, a target, and a white line of 2.3 mm width and a length of 90 mm. The experimenter rotated its orientation by way of pressing a key, beginning at the direction opposite the respective target. The task of the participant was to instruct the experimenter to rotate the line around the start location until it matched the direction of the hand movement he or she thought appropriate to move the cursor from the start circle to the target circle.

In the proprioceptive-shift test, participants had to perform eight periodic back-and-forth or right-and-left movements in response to vertical (A) or horizontal (B) arrows presented on the screen in the order ABBAABBA. Movements were paced by a computer-generated tone at a frequency of 1 Hz, i.e. participants were instructed to complete one movement per second in a particular direction. For the visual-shift test, participants were instructed to align two filled white circles of 4.8 mm diameter, which marked the ends of an invisible line being rotated around the center of the screen, with the vertical or horizontal (trial order: VHHVVHHV). Participants instructed the experimenter to rotate the endpoints of the invisible line until they were aligned with what participants perceived to be the horizontal or vertical axis on the screen. Both the proprioceptive-shift test and the visual-shift test were preceded by 8 maintenance trials that were identical to baseline trials.

Subsequent to baseline practice and pretests, the visuo-motor transformation was practiced for 10 blocks, each with 48 visual closed-loop trials. The cursor was visible as during baseline practice, but the start location was colored red to cue the presence of the rotation, and an additional hand target was presented at the target amplitude of 90 mm and shifted 75° CCW relative to the cursor target. Participants had been informed about the meaning of the color of the start circle in terms of the absence and presence of a rotation, but not about its size and direction.

The practice phase was followed by five different posttests, an open-loop test with cued presence of the transformation, an open-loop test with cued absence of the transformation, an explicit test with cued transformation, and the tests of visual shift and proprioceptive shift. The open-loop test with cued presence of the transformation differed only with respect to the color of the start circle from the open-loop test without transformation. The open-loop test with cued absence of the transformation was identical to the open-loop pretest. Of course, in the maintenance trials, which preceded each block of test trials, the transformation was present and the start circle was red. In the explicit test, which was otherwise identical to the explicit pretest, the presence of the transformation was cued.

On the second day each participant repeated the procedure of the first day. After another baseline phase with a rotation of 0°, which also served as a washout phase to avoid potential carry-over effects from day 1, along with the respective pretests, a visuo-motor rotation of 75° CCW rather 75° CW was used. This time, there was no hand target presented on the screen during practice. The major purpose of this addition to the main part of the experiment was to check the robustness of previous findings [20], which serve as a reference to assess the qualitative effects of the additional hand target presented during practice.

Each single trial started with the presentation of the start circle. Its color was red or green depending on whether the presence or absence of the visuo-motor transformation was cued. The cursor appeared on the monitor when it entered a tolerance range of 15.2 mm around the center of the start circle. It was presented to assist participants in homing-in on the start position. When the cursor was within a tolerance range of 2 mm around the center of the start circle for 500 ms, a tone (1000 Hz, 26 ms) was presented and the start circle was filled. For a randomly chosen period of 500, 700, 900, 1100, or 1300 ms the finger had to remain in the start location, otherwise the trial was reset. At the end of this waiting period a target appeared. Simultaneously the start circle disappeared and subjects could start their movement.

The end of the movement was determined online by a velocity criterion, provided that the cursor had left the tolerance range of 15.2 mm around the center of the start circle. The velocity criterion required that the distance between successively sampled positions was not larger than 0.25 mm for more than 400 ms. Only in visual closed-loop trials there was an accuracy criterion in addition, in that the deviation from the target position had to be less than 3 mm. If the target was not reached within 5 seconds after movement initiation, a buzzer sound was presented signaling abortion of the trial and a time-out message was displayed in the center of the screen. Upon the end of a trial, cursor and target disappeared. The movement back to the start location was always open-loop except for the final homing-in. To assist in finding the start location, arrows were presented on the screen, which indicated the start position relative to the current position of the hand.

### Data analysis

For each trial the x and y positions of both the finger on the table and the cursor on the monitor were recorded, with the start positions as the origins of the respective Cartesian coordinate systems. Each of the resulting time series was low-pass filtered (fourth-order Butterworth, 10 Hz, dual pass) and differentiated (two-point central difference algorithm). Start and end of the movements were determined based on tangential velocity of the finger. Starting from peak tangential velocity, both in a forward and a backward search those samples were determined at which tangential velocity was less than 5 mm/s for the first time and remained smaller for 200 ms thereafter.

Practice and test trials were analyzed in terms of three parameters, movement time and errors of terminal and initial direction. The direction of the vector from the start position to the endpoint defined terminal direction. Initial direction was defined as the direction after a movement duration of 200 ms. All directional variables used for further analyses were expressed relative to target directions, so that normal averaging procedures rather than circular means could be used.

For each block of practice trials and each test phase means were computed for each participant across all visual target directions following a screening for irregular trials. Movements with a duration of less than 200 ms were considered irregular, as were movements for which the total trajectory was longer than 5 times the target amplitude. In addition, in the closed-loop practice phases with and without the visuo-motor rotation, aborted trials (those that were not finished within 5 seconds) were reanalyzed. All of these trials, which ended with the cursor position within a 6-mm radius around the center of the respective target, were also included in subsequent analyses.

On the first day, 705 of 15552 trials (4.5%) were excluded from further analyses in the younger group, and 514 of 15552 trials (3.3%) in the older group. On the second day, 467 of 15552 trials (3.0%) were classified as irregular in the younger group, and 401 of 15552 trials (2.6%) were excluded in the older group.

For the open-loop tests and the explicit judgments, posttest minus pretest differences of hand directions were computed. For the tests with cued visuo-motor transformation (red start circle) these differences are designated as adaptive shifts, for the tests with cued absence of the transformation they are designated as aftereffects, and for the explicit tests as explicit shifts.

For the visual-shift test, pretest-to-posttest changes in deviations from the horizontal and vertical axes were calculated which represent the visual shifts. For proprioceptive-shift tests, the continuous periodic movements were parsed into movements from right to left and left to right or from front to back and back to front. For each movement, principal component analysis was used to determine its main orientation. Changes in the mean deviations of these orientations from the x and y axes, respectively, from pre- to posttest are referred to as proprioceptive shifts.

## Results

Results will be reported for the two days of the experiment, first for the practice phases with the transformation present on day 1 and day 2, and second for the pretest-to-posttest changes. The Greenhouse-Geisser epsilon [45] was evaluated to determine whether the repeated measures data met the assumption of sphericity (Σ>0.75). In cases where sphericity was not met, the *F* statistic was evaluated for significance using the Greenhouse–Geisser adjusted degrees of freedom, though the uncorrected degrees of freedom are reported.

### Practice phases

In the practice trials visual feedback was presented. In addition, movements had to be accurate in order for the trials to be ended. Therefore errors of terminal direction were negligible, and the analysis was restricted to the initial-direction errors and movement times.

**Figure 1:**
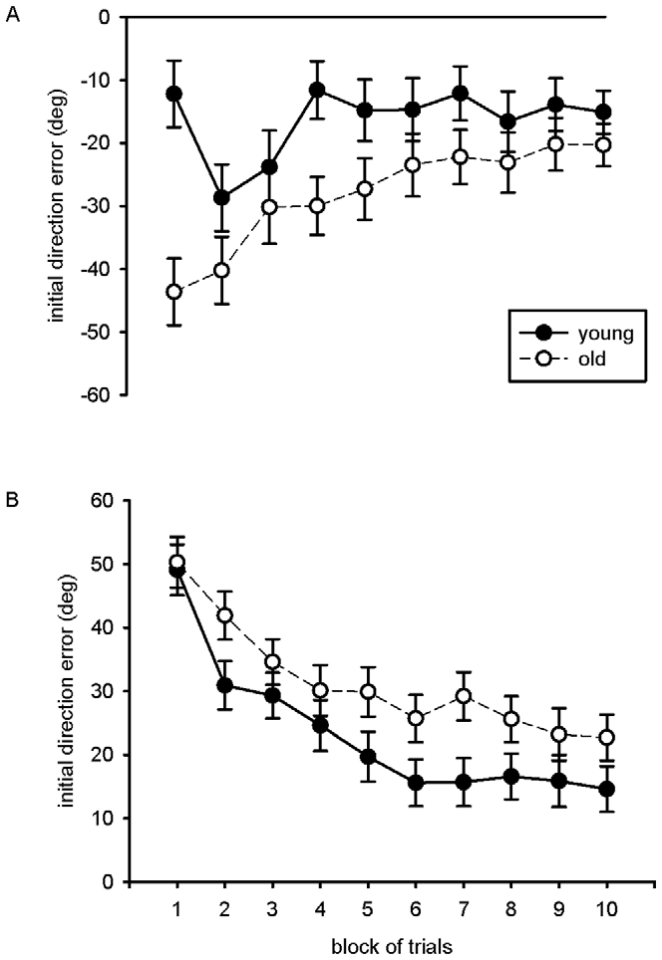
Mean initial errors of cursor direction (200 ms after movement onset) during practice of the young and old group as a function of block of trials. (a) Day 1 with additional hand target and 75° CW rotation, (b) day 2 without augmented information and 75° CCW rotation (error bars indicate standard errors of the mean).

In Figure 1 the mean errors of initial direction in the practice phases of day 1 and 2 are shown. These are deviations of the cursor direction on the monitor from the visual target direction at a movement duration of 200 ms. On the first day, they were −12.2° and −15.2° in the first and last block of practice for young participants and −43.5° and −20.3° for old participants, respectively. Young participants initially had small errors, which increased in the second block of trials and declined again. The older participants, in contrast, exhibited a rather continuous decline of errors, which nevertheless were overall larger than in the young participants. A two-way ANOVA with the between-participant factor age and the within-participant factors block and target direction revealed a significant main effect of age, *F*(1,34)  = 4.5, *p*<.05, a significant main effect of block, *F*(9,306)  = 7.3, *p*<.01, and a significant interaction of these two factors, *F*(9,306)  = 3.5, *p*<.01.

On the second day, initial-direction errors improved in the course of practice from 49.7° in the first block of trials to 18.7° in the last block, *F*(9,306)  = 25.4, *p*<.01. Averaged across target directions, the errors were larger for the old than for the young participants, *F*(1,34)  = 4.1, *p*<.05. This group difference developed early in practice and was present throughout the whole practice phase with the interaction of age and block being not significant, *F*<1.

**Figure 2:**
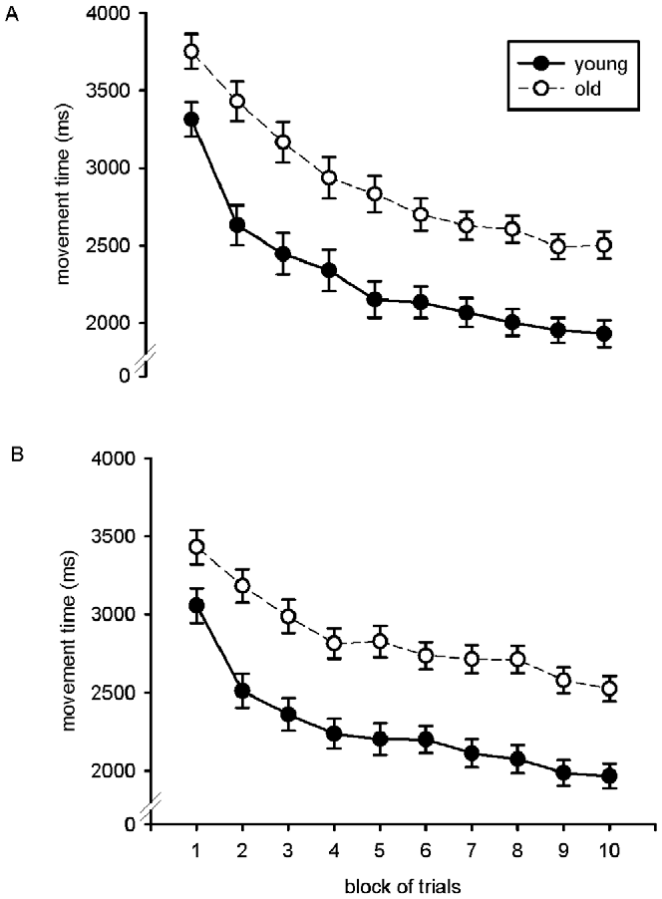
Mean movement time during practice of the young and old group as a function of block of trials. (a) Day 1 with additional hand target and 75° CW rotation, (b) day 2 without augmented information and 75° CCW rotation (error bars indicate standard errors of the mean).

The mean movement times in the practice phase are shown in Figure 2. Movement time on the first day was 2297 and 2904 ms for young and old participants, respectively, *F*(1,34)  = 20.9, *p*<.01. It declined in the course of practice, *F*(9,306)  = 99.5, *p*<.01, with means of 3123 ms in the first three blocks of trials and 2248 ms in the last three blocks. The interaction of block and age failed to approach statistical significance, *F*(9,306)  = 1.4. *p*>.1.

On day 2, movement time was 2272 and 2851 ms for the young and old participants, respectively, *F*(1,34)  = 23.4, *p*<.01. It declined in the course of practice, *F*(9,306)  = 77.7, *p*<.01, with means of 2922 ms in the first three blocks of trials and 2308 ms in the last three blocks. This decline was more pronounced in the younger group (ΔMT = 1090 ms from first to a last block) as compared to the older group (ΔMT = 906), but the interaction age x block failed to approach significance, *F*(9,306)  = 1.5, *p*>1.

### Tests

For the statistical analyses of the adaptive shifts, aftereffects, and explicit shifts, individual posttest-pretest differences of the terminal directions of hand movements were calculated and subjected to a series of ANOVAs. For each type of test, open-loop with cued transformation, open-loop with cued absence of the transformation, and explicit test with cued transformation, the ANOVA included the between-participant factor age. Posttest-pretest differences were also computed for visual and proprioceptive shifts and subjected to ANOVAs with the between-participant factor age and the within-participants factor movement direction (forward-backward vs. left-right).

The analysis of pointing performance in the open-loop pretests revealed a significant difference between age groups only on the day 2, *F*(1,34)  = 9.4, p<.01. However, this difference amounted to only 2.1°, which seems negligible for the calculation of the pretest-to-posttest differences. Furthermore, the deviation in movement direction of the younger group was negative, which would only have served to decrease the pre-to-posttest differences in case of adjusting to a counterclockwise visuo-motor rotation as employed on day 2. The analysis of explicit judgments of pointing direction in the pretest on both days revealed no significant differences, *F*<1, and *F*(1,34)  = 1.5, *p*>.2, respectively. In addition, there were no age-related differences in visual or proprioceptive pretest performance (all *F*s<1).

**Figure 3:**
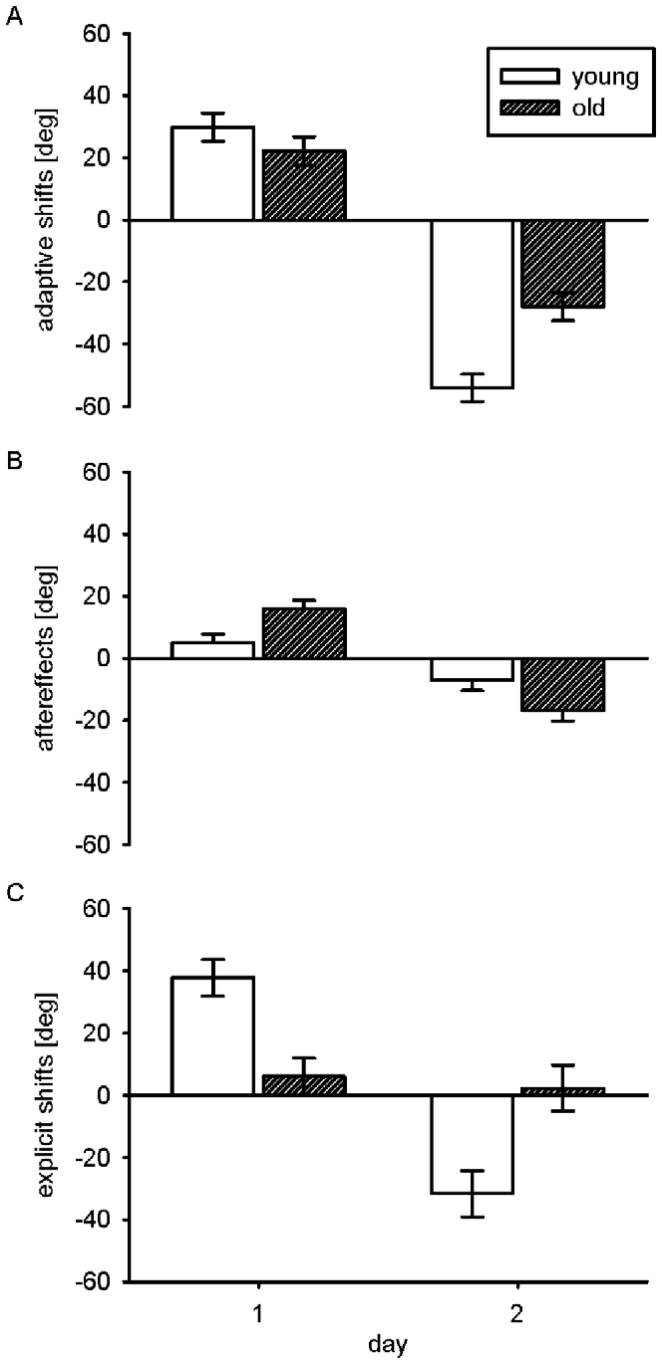
Pre-to-posttest changes in terminal movement direction relative to the visual target direction. Mean (a) adaptive shifts, (b) aftereffects, and (c) explicit shifts in visual open-loop tests with cued presence (a, c) and cued absence (b) of the transformation for the young and old group averaged across target directions shown separately for day 1 after practice of the 75° CW rotation with the additional hand target present and for day 2 after practice of the 75° CCW rotation without augmented information (error bars indicate standard errors of the mean).

For the open-loop posttest with cued presence of the visuo-motor rotation an adaptive shift of +75° would compensate the visuo-motor rotation of −75° on the first day and vice versa on the second day. The mean adaptive shifts are shown in Figure 3a. Averaged across target directions, adaptive shifts on day 1 were different from zero both for the young and for the older participants. For the young participants they were 29.8°, *F*(1,34)  = 42.4, *p*<.01, and for the old participants they were 22.2°, *F*(1,34)  = 23.6, *p*<.01. The difference between the two age groups failed to reach statistical significance, *F*(1,34)  = 1.4, *p*>.2.

In contrast to the adaptive shifts observed after practice with the additional hand target, adaptive shifts on the second day were consistently larger for the young participants giving rise to a significant main effect of age, *F*(1,34)  = 17.6, *p*<.01. For both age groups they were significantly different from zero, −54.0°, *F*(1,34)  = 151.7, *p*<.01, and −28.0°, *F*(1,34)  = 40.8, *p*<.01, respectively.

The visual open-loop tests with cued absence of the visuo-motor rotation served to assess aftereffects. Their means are shown in Figure 3b. On the first day with the additional hand target present, aftereffects were stronger for the old than for the young participants, *F*(1,34)  = 8.0, *p*<.05. For the old participants they were 16.0°, which differed significantly from zero, *F*(1,34)  = 33.8, *p*<.01, whereas aftereffects of the young participants with a mean of 5.0°, were not significantly different from zero, *F*(1,34)  = 3.3, *p*<.1.

As on the first day, the aftereffects on day 2 appeared slightly larger for the old participants, but this age effect failed to reach statistical significance, *F*(1,34)  = 4.0, *p*<.1. Averaged across target directions, aftereffects in both groups were significantly different from zero. They were −7.0° for the young, F(1,34)  = 4.1, *p* = .05, and −16.7°, *F*(1,34)  = 23.7, *p*<.01, for the old group.

The mean shifts of explicit judgments are shown in Figure 3c. Despite the continuous presence of the additional hand target on the screen during practice, older participants exhibited a smaller systematic shift of explicit judgments in the cued presence of the visuo-motor rotation than the young participants. Averaged across directions, the shift of explicit judgments did not differ significantly from zero for the old group (6.1°, *F*(1,34)  = 1.1, *p*>.3), whereas it did so for the young group (37.8°, *F*(1,34)  = 39.7, *p*<.01). The difference between the age groups was significant, *F*(1,34)  = 13.9, *p*<.01.

On the second day, there was a highly significant main effect of age, *F*(1,34)  = 10.5, *p*<.01. For the young participants the mean explicit shift, −31.7°, was significantly different from zero, *F*(1,34)  = 18.1, *p*<.01, whereas for the old participants, 2.3°, this was not the case, *F*<1.

**Figure 4:**
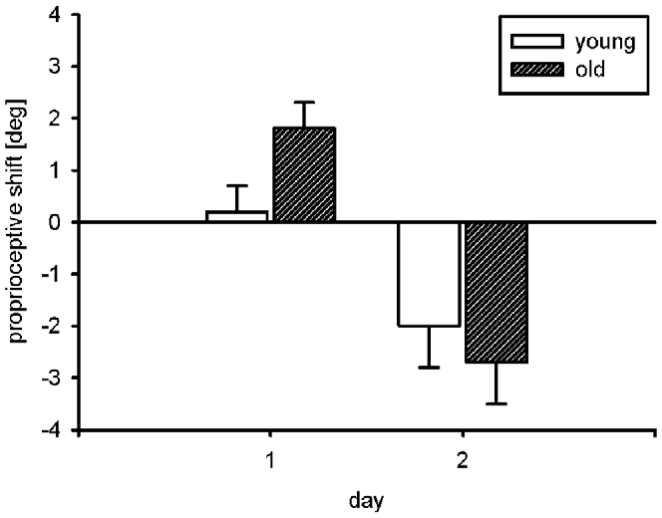
Mean proprioceptive shifts in the young and old group on both days of the experiment (error bars indicate standard errors of the mean).

The test of visual shifts revealed no changes from pretest to posttest, neither for the young nor for the old participants. The mean proprioceptive shifts are shown in Figure 4. Whereas for the young participants the proprioceptive shift on day 1 was negligible, 0.2°, and not significantly different from zero, *F*<1, for the old participants it was small, 1.8°, as compared to the visuo-motor rotation of −75°, but nevertheless significant, *F*(1,34)  = 14.3, *p*<.01. The difference between the two age groups was significant as well, *F*(1,34)  = 5.6, *p*<.05.

On day 2, as on the first day, there was no visual shift in either group. However, both groups exhibited a systematic proprioceptive shift in the clockwise direction, as shown in Figure 4. It was −2.0° for the young and −2.7° for the old participants. Both shifts were significantly different from zero, *F*(1,34)  = 5.7, *p*<.05, and *F*(1,34)  = 10.4, *p*<.01, respectively, and not significantly different from each other, *F*<1.

### Adaptive shifts, aftereffects, and explicit judgments

In order to assess the role of explicit knowledge for the adaptive shifts and aftereffects, we classified participants as having no, intermediate or full explicit knowledge of the transformation. For a participant to be classified as having full explicit knowledge, his or her explicit shift, averaged across directions, had to be larger than 60°, whereas all participants with mean shifts smaller than 15° were classified as having no explicit knowledge.

Fourteen older and 5 younger participants with no explicit knowledge of the visuo-motor rotation of −75° CW were compared. Neither for adaptive shifts nor for aftereffects the difference between young and old participants was significant, 14.8° vs. 22.3°, *F*(1,17)  = 1.0, *p*>.2, and, 7.4° vs. 16.1°, *F*(1,17)  = 1.3, *p*>.2, for adaptive shifts and aftereffects, respectively. In order to exclude the possibility of non-significant age effects simply because of low statistical power due to a small number of cases in the younger group, we did a second set of ANOVAs involving the fourteen older participants without explicit knowledge and the five younger participants with full explicit knowledge. These ANOVAs involved the same group sizes as the previous comparison, but the two age groups differed in explicit knowledge rather than being comparable in this respect. The results showed a significant main effect of age for the adaptive shifts, 42.7° vs. 22.3°, *F*(1,17)  = 5.0, *p*<.05, but not for the aftereffects, 6.7° vs. 16.1°, *F*(1,17)  = 1.6, *p*>.2.

For the same analysis on the second day, the cutoff for full explicit knowledge was chosen somewhat smaller than for the first day to obtain a sufficiently large sample of participants with perfect explicit knowledge. The respective cutoff for being classified as having no explicit knowledge was set to −15°, whereas all participants with explicit shifts of at least −45° were classified as having full explicit knowledge. This procedure yielded 5 young and fifteen old participants with no explicit knowledge of the transformation. When these two groups were compared, the age effect on the adaptive shifts was only marginally significant, -41.8° vs. −22.5°, *F*(1,18)  = 3.9, *p*<.1. There was no age-related variation of aftereffects, −11.0° vs. −10.6°, *F*<1. The ANOVAs involving the 5 younger participants with full explicit knowledge showed a highly significant main effect of age for the adaptive shifts, −66.1° vs. −22.5°, *F*(1,18)  = 27.8, *p*<.01. Aftereffects turned out to be somewhat smaller for young participants with explicit knowledge than for old participants without explicit knowledge, −6.3° vs. −10.6°, but this difference failed to approach significance, *F*(1,18)  = 2.8, *p*>.1.

## Discussion

The purpose of the present study was examine a means to boost the acquisition of explicit knowledge about a visuo-motor transformation in particular in older adults. The reason for doing so was the well-supported hypothesis that age-related variations of adjustment to visuo-motor rotations are due to differences in the acquisition of explicit knowledge and/or in the use of strategic corrections based on this knowledge [11]–[14], [16].

Our means to boost the acquisition of explicit knowledge of the transformation was based on a rather straightforward consideration. The nature of a simple kinematic transformation such as a visuo-motor rotation becomes obvious when not only the target for the output signal, the position of the cursor, is presented, but also the target for the input signal, the position of the hand. This was realized in the present study, except for the fact that the target position for the hand was presented on the monitor rather than in the plane of the hand movement. The difference between these planes should not matter given the high accuracy with which the directions of hand movements can be matched to directions presented on the monitor [16].

All in all, we were able to replicate the previously reported age-related difference in explicit knowledge and its functional relation to an increase in adaptive adjustments via strategic corrections [16], [18] on day 2, but unexpectedly also on day 1 despite the presence of the augmented information. Thus, the presentation of the additional hand target failed to enhance explicit knowledge. In the group of older participants, on the average no explicit knowledge at all was acquired, as it was also the case on day 2 and in a previous study [16] after practice without the augmented information. Regarding the adaptive shifts and aftereffects, the augmented information had primarily the effect of making the data appear noisier. For the adaptive shifts the typical age difference was present, but not statistically significant as it had been on day 2 and in the previous study [16]. In contrast, the larger aftereffects of the older participants, which had been present, but non-significant, on day 2 and in the previous study, were significantly stronger after practice with the augmented information. These undulations of the age-related variations around the threshold of statistical significance might be chance results.

Whereas the augmented information failed to affect the age-related variations of explicit knowledge, adaptive shifts, and aftereffects in a substantial way, it produced clear age-related effects on initial direction errors during practice and on proprioceptive shifts. During practice accurate performance was possible because of continuously available visual feedback of the cursor. Typical for adjustment to an abruptly introduced visuo-motor rotation are the curved paths of the cursor early in practice [16], with initial-direction errors of the cursor in the same direction as the visuo-motor rotation. With the augmented information, in principle, even the initial direction error can be compensated right from the start of practice in that the hand movement is directed to the additional hand target.

Contrary to expectations, older participants exhibited a similar pattern of initial direction errors in the course of practice on both days, i.e. irrespective of the presence or absence of the additional hand target. While younger participants also showed a gradual reduction of initial direction errors during practice on day 2, they exhibited a different pattern when the augmented information was present on the first day. They started with small errors of initial direction, but these errors showed a sudden increase in the second block of practice being slowly reduced thereafter, with the further practice curve being basically parallel to the practice curve of the older participants. Thus, whereas the young participants seemed to make use of the additional hand target early in practice on day 1, this was apparently not the case for the older participants.

A possible reason for the older adults' decision to ignore the additional hand target might lie in the nature of our task. Participants were given five seconds to reach the target. Thus, it was possible to successfully complete the task without using the additional hand target at the expense of curved rather than straight movements. Furthermore, it could be argued that there was a certain pressure not to make use of the augmented information. Moving to two targets simultaneously, to the one with the cursor and to the other with the hand, can be conceived as a dual-task, even though the two targets are related in a contingent way. The costs of dual-task performance are likely to be higher for older than for younger adults [46]. So for older participants the costs of using or the benefits of not using the augmented information should be larger, and indeed the present data indicate that they neglected the augmented information completely.

In a previous study, Mazzoni and Krakauer [22] reported an overcompensation of rotation in the presence of an additional hand target for directional errors during practice. Overcompensation means that in their study the cursor tended to move towards the hand target, whereas in our study the hand tended to move towards the cursor target. Mazzoni and Krakauer interpreted their pattern of results as evidence for the superposition of explicit and implicit adjustments, in which the implicit adjustment is added to the explicit strategy yielding an overcompensation of the rotation. The discrepancy between our findings and those of Mazzoni and Krakauer [22] sheds doubt on this interpretation. Again, if we conceive the current task as a dual-task, it would contain movements of two end effectors towards two separate targets, a proprioceptive target and a visual target. The priority, which is assigned to these two movement goals, varies as a function of task demands. Mazzoni and Krakauer used rapid reversal movements, the accuracy of which is likely to depend on initial direction towards the proprioceptive hand target, whereas we used discrete movements with rather broad time constraints that were required to end right on the visual target. Thus, by increasing the accuracy demands of the cursor movement endpoint and permitting visual closed-loop control to achieve this, the visual goal became more important, so that movements tended towards the cursor target. Thus, Mazzoni and Krakauer [22] might not have tapped explicit and implicit processes of visuo-motor adjustment, but interference between pointing to two targets simultaneously instead. Of course, this is a hypothesis rather than an explanation, which requires further experimental study.

In order to gain further insights in the effects of the additional hand target on implicit processes of adaptation, we also examined visual and proprioceptive shifts even though in adaptation to an extrinsic visuo-motor rotation, there is no sensory discordance in a strict sense because vision and proprioception refer to different objects. It is not clear, however, whether sensory discordance, as present in prism adaptation, but absent with extrinsic transformations, is indeed a prerequisite for such changes to occur.

Augmented information abolished the small, but reliable proprioceptive shifts in young adults, but not in old adults. Before we outline possible reasons for this, it is remarkable that there were proprioceptive shifts at all, though no visual shifts. The only study in which proprioceptive shifts after adaptation to a visuo-motor rotation have been examined has been reported by Wong and Henriques [47], and in that study they were absent. There are several reasons for this discrepancy. For example, Wong and Henriques [47] assessed proprioceptive shifts in terms of judgments of the inclination of a felt contour rather than in terms of the matching of instructed movement directions. In addition, they introduced the visuo-motor rotation in small steps, so that it remained basically unnoticed by the participants. Perhaps most important, their visuo-motor rotation amounted to only 30°. Given that we observed shifts of only about 2° after fairly long practice with a 75° rotation, it seems not unlikely that the smaller expected shifts for a 30° rotation might be missed by statistical tests.

In spite of the finding of proprioceptive shifts, there were no visual shifts. There was an important difference between the tests we used that might be responsible for this difference: the visual tests required judgments of horizontal and vertical in an allocentric frame of reference provided by the computer monitor and the visible surroundings. In contrast, the proprioceptive task required forward-backward and left-right movements in a basically egocentric frame of reference.

Proprioceptive shifts were absent only in young participants after practice with the augmented information. Perhaps the presentation of the hand target that had to be reached with the invisible hand had the effect of directing attention to the feel of the hand movements. Generalizing from prism-adaptation studies [30]–[33], proprioceptive shifts should be reduced under such conditions. In the older group of participants, the practice data gave no evidence that the supplementary hand targets had been used at all to direct the hand. Therefore attention to proprioceptive information was not enhanced, and a proprioceptive shift was present as in conditions without augmented information. Thus, the specific absence of proprioceptive shifts lends further support to the notion of age-related differences in the usage of the additional hand target during visuo-motor adjustment.

Based on the present data, future research will evaluate the hypothesis that the concurrent presence of targets for both, the body end effector and the effective part of a tool (here, the cursor) interfere with each other because of dual-task demands and address alternative ways of providing augmented information to boost explicit knowledge and facilitate strategic corrections during adaptation to novel visuo-motor mappings in the elderly. Given the technological advances in the design of modern tools and the aging workforce, especially the latter is of interest for practical applications such as the design of age-differentiated workplaces or the development of training procedures, for instance for laparoscopic surgery. Our suggestion is to use augmented information that can be integrated in the task at hand. Possible manipulations to achieve this range from increasing the transparency of the visuo-motor transformation – for instance by presenting a visual depiction of the transformation instead of just its output or by increasing the acuity of the visual and proprioceptve feedback - to the employment of robot-generated assistance patterns, which will be addressed in future research.
